# Functional and Transcriptional Responses of Neutrophils from Dogs Living Under Different Housing Conditions Following In Vitro Lipopolysaccharide Stimulation

**DOI:** 10.3390/ijms27188112

**Published:** 2026-09-11

**Authors:** Marek Kulka, Iwona Monika Szopa, Bartłomiej Różycki, Maciej Klockiewicz

**Affiliations:** 1 Department of Pathology and Veterinary Diagnostics, Institute of Veterinary Medicine, Warsaw University of Life Sciences, 02-776 Warsaw, Poland; 2 Department of Physiological Sciences, Institute of Veterinary Medicine, Warsaw University of Life Sciences, 02-776 Warsaw, Poland; iwona_szopa@sggw.edu.pl; 3 Veterinary Outpatient Clinic “ANDA”, 05-510 Konstancin-Jeziorna, Poland; 4 Department of Preclinical Sciences, Institute of Veterinary Medicine, Warsaw University of Life Sciences, 02-786 Warsaw, Poland; maciej_klockiewicz@sggw.edu.pl

**Keywords:** antigen stimulation, canine leukocytes, cytokines, CXCR1, CXCR2

## Abstract

Environmental conditions have an impact on animals’ immune systems. Neutrophils, as key components of innate immunity, rapidly respond to bacterial stimuli through the activation of inflammatory and migratory pathways. This study evaluated the transcriptional and functional responses of neutrophils derived from dogs living under different housing environments following in vitro lipopolysaccharide (LPS) stimulation. Distinct transcriptional profiles were observed among groups in genes associated with leukocyte adhesion (*CD11b*), chemokine signaling (*CXCR1*, *CXCR2*, and *CXCL8*), and cytokines (*TNF-α*, *IL-1β*, and *IL-6*). Additional cytokine analyses confirmed neutrophil activation and revealed differences in the magnitude and duration of cytokine secretion between groups. These findings indicate that environmental background may be associated with differences in both transcriptional responsiveness and cytokine production in canine neutrophils. Dogs originating from different housing conditions exhibited distinct patterns of inflammatory activation. The combined assessment of gene expression and cytokine secretion provides valuable insight into innate immune adaptation and might represent a useful approach for monitoring immune status in dogs.

## 1. Introduction

Innate immunity represents the first line of defense against environmental and microbial challenges. Neutrophils and monocytes are key effector cells of this system, rapidly responding to pathogens through receptors such as Toll-like receptors (TLRs) [1,2,3]. Upon activation, these cells initiate transcriptional programs that lead to the production of pro-inflammatory cytokines and chemokines, thereby orchestrating local and systemic immune responses [4]. Lipopolysaccharide (LPS), a major structural component of the outer membrane of Gram-negative bacteria, is a potent activator of innate immune cells [2].

Binding of LPS to Toll-like receptor 4 (TLR4) triggers intracellular signaling pathways, ultimately resulting in the modulation of genes encoding key inflammatory mediators and molecules involved in leukocyte trafficking [2,5,6]. Among these are pro-inflammatory cytokine genes, including interleukin 6 (IL-6), interleukin 1 beta (IL-1β), and tumor necrosis factor alpha (TNF-α), as well as the chemokine CXCL8 (IL-8) [7,8,9]. In addition to these mediators, LPS stimulation modulates the expression of chemokine receptors such as CXCR1 and CXCR2, which regulate neutrophil responsiveness to CXCL8 gradients, and adhesion-related molecules including CD11b and CD18 (forming the β2 integrin Mac-1 complex) and CD62L (L-selectin), which are critical for leukocyte rolling, firm adhesion, and transendothelial migration [9,10,11,12,13]. Evaluation of genes encoding these molecules provides a comprehensive view of the transcriptional regulation of inflammatory signaling and migratory capacity in innate immune cells. Transcriptional activation is functionally reflected in the production and secretion of effector cytokines, including TNF-α, IL-1β, IL-6, and CXCL8. These cytokines play central roles in amplifying inflammation, recruiting additional immune cells, and shaping the downstream adaptive immune response [4,7]. Therefore, LPS stimulation in vitro provides a controlled model to evaluate the functional and transcriptional responsiveness of leukocytes under conditions mimicking bacterial exposure. The immune response may differ based on various environmental factors [14,15]. Dogs living under different housing conditions are exposed to distinct combinations of stimuli, including differences in daily routines, social interactions, management practices, population density, and exposure to microorganisms and antigens [14]. These factors may contribute to shaping immune homeostasis and influence the functional responsiveness of circulating leukocytes, including neutrophils, by modulating inflammatory signaling, cytokine production, and cellular activation [16]. As neutrophils constitute the first line of defense against pathogens, alterations in their responsiveness may reflect adaptive changes in innate immune function associated with different living conditions [13]. Therefore, comparing leukocyte responses, particularly those of neutrophils, between dogs living under different housing conditions may provide valuable insight into variation in innate immune responsiveness.

The study aimed to evaluate the transcriptional activity and cytokine production profile of canine neutrophils stimulated in vitro with LPS. We assessed gene expression profiles encompassing chemokine receptors (*CXCR1* and *CXCR2*) and adhesion-related molecules (*CD11b*, *CD18*, and *CD62L*), as well as genes encoding pro-inflammatory cytokines (*IL-6*, *IL-1β*, and *TNF-α*) and chemokines (*CXCL8*), together with their corresponding protein secretion. We hypothesized that different environmental conditions influence transcriptional responsiveness and cytokine production following LPS stimulation, reflecting changes in immune adaptation and providing insights into animal welfare.

## 2. Results

### 2.1. CD11b Expression

At baseline, client-owned dogs exhibited significantly higher *CD11b* mRNA levels than short-term (*p* < 0.01) and long-term shelter dogs (*p* < 0.05). Following 3 h of stimulation, *CD11b* expression was significantly higher in short-term stay dogs compared to both long-term (*p* < 0.05) and client-owned (*p* < 0.01) groups. No significant between-group differences were observed after 15 h of stimulation.

**Figure 1:**
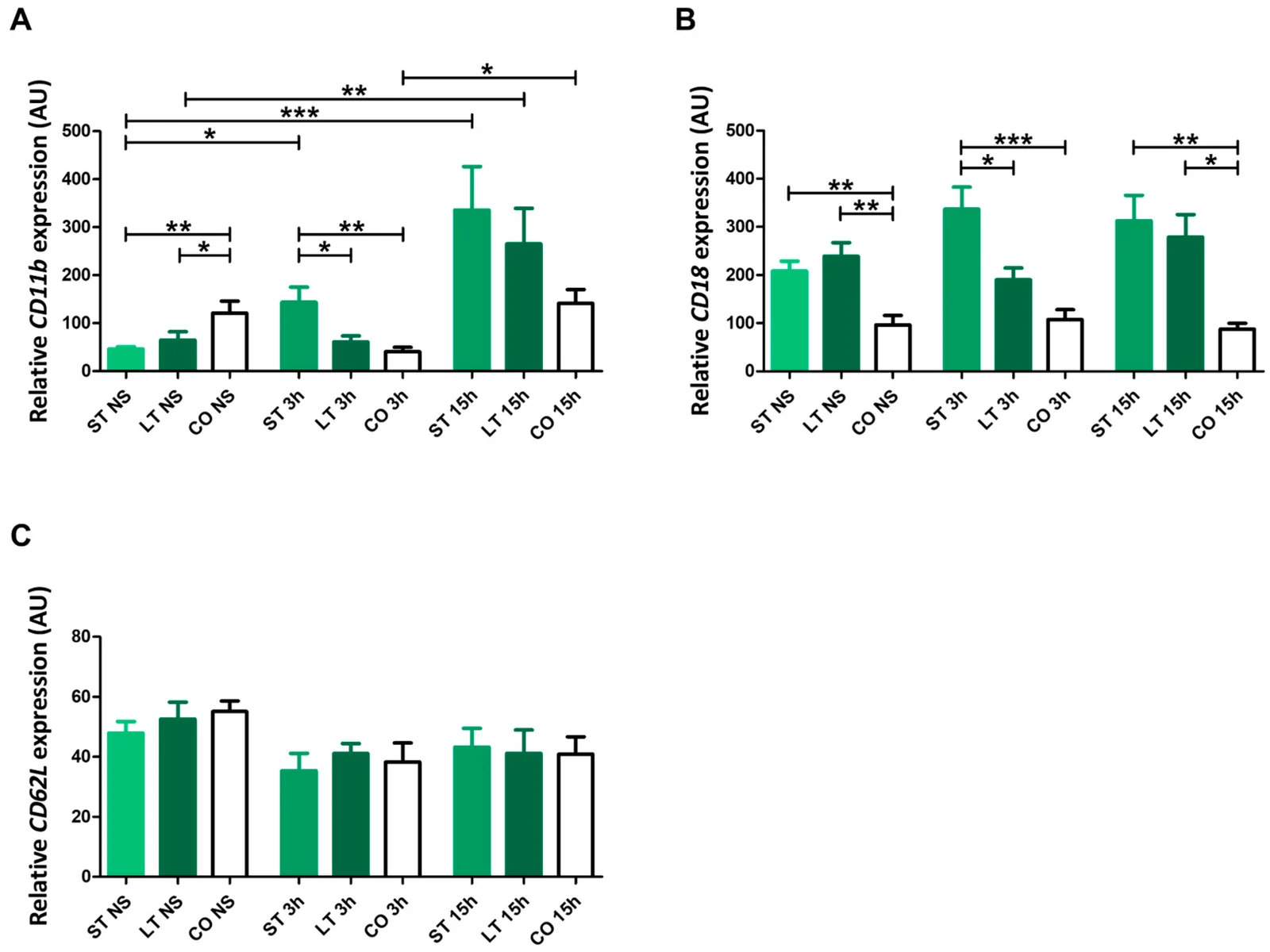
Expression of leukocyte adhesion-related genes. Bar graphs showing relative mRNA expression (arbitrary units, AU) of *CD11b* (**A**), *CD18* (**B**), and *CD62L* (**C**) in cells isolated from short-term (ST), long-term (LT), and client-owned (CO) dogs at baseline (non-stimulated cells, NS) and after 3 h and 15 h of LPS stimulation. Data are shown as mean results (*n* ≥ 6), and error bars indicate SEM. Statistical analysis was performed by one-way analysis of variance (ANOVA) with Tukey’s multiple comparisons test or the Kruskal–Wallis test followed by Dunn’s multiple comparisons test (* *p* < 0.05, ** *p* < 0.01, *** *p* < 0.001).

Within-group analysis revealed a significant response in *CD11b* expression at 3 h and 15 h compared with baseline in short-term stay dogs (*p* < 0.05 and *p* < 0.001, respectively). In long-term shelter dogs, a significant increase was observed only after 15 h (*p* < 0.01). Additionally, *CD11b* expression at 15 h was significantly higher than at 3 h in client-owned dogs (*p* < 0.05). Overall, these findings suggest a time-dependent increase in *CD11b* mRNA expression following antigenic stimulation, with the most pronounced differences observed during the early phase of activation (Figure 1A).

### 2.2. CD18 Expression

Client-owned dogs exhibited significantly lower *CD18* mRNA expression than both short- and long-term stay dogs at baseline (both *p* < 0.01) and after 15 h of stimulation (*p* < 0.01, *p* < 0.05, respectively). After 3 h of stimulation, *CD18* expression was significantly higher in short-term stay dogs than in both long-term (*p* < 0.05) and client-owned (*p* < 0.001) groups (Figure 1B).

### 2.3. CD62L Expression

No significant differences in *CD62L* mRNA expression were observed among the study groups at baseline, 3 h, or 15 h following stimulation. Similarly, within-group analyses revealed no significant changes in *CD62L* expression over time in any of the experimental groups (Figure 1C). Additional analysis by flow cytometry at baseline revealed a similar pattern of surface CD62L expression, with the lowest levels observed in short-term stay dogs, showing a statistically significant difference compared to client-owned dogs (*p* < 0.01) (Supplementary Figure S1).

### 2.4. CXCR1 and CXCR2 Expression

*CXCR1* and *CXCR2* expression showed a similar pattern. At baseline, client-owned dogs exhibited significantly higher mRNA levels of both *CXCR1* and *CXCR2* compared to short-term (both *p* < 0.001) and long-term stay dogs (*p* < 0.01, *p* < 0.001 for *CXCR1* and *CXCR2,* respectively). Following LPS stimulation, significant differences were observed only after 3 h, when short-term shelter dogs showed higher *CXCR1* and *CXCR2* expression compared to client-owned dogs (both *p* < 0.05).

**Figure 2:**
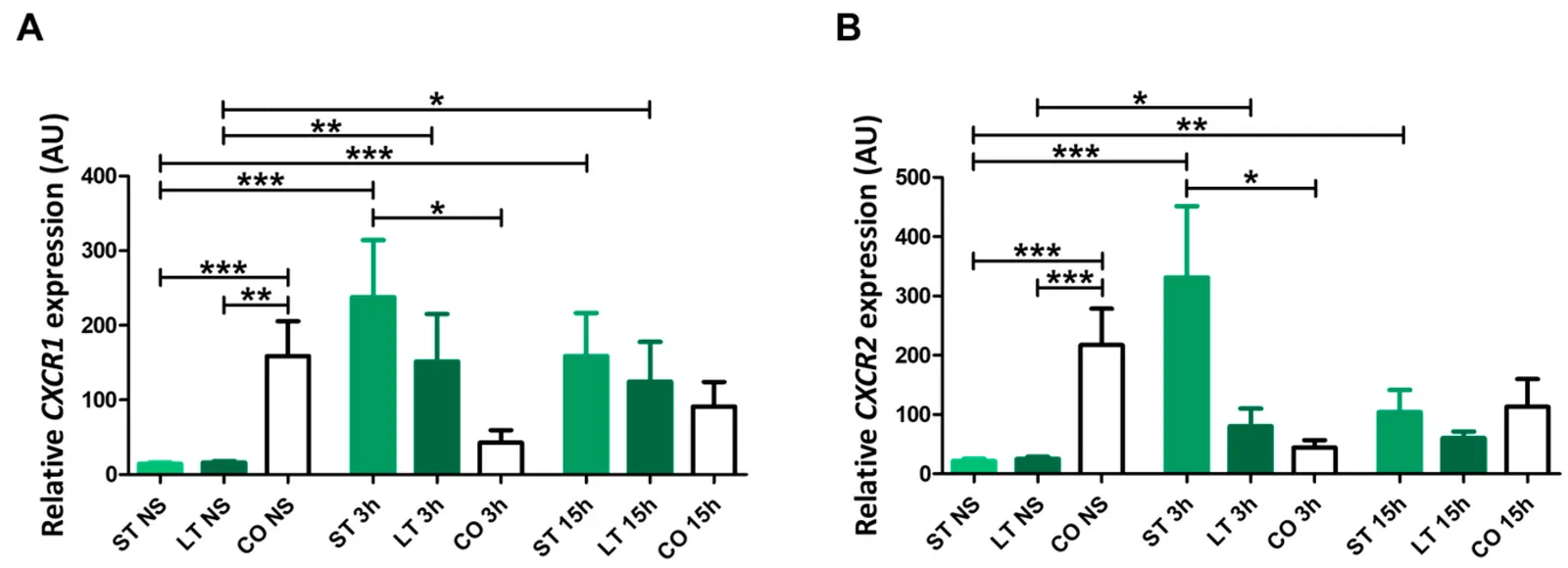
Expression of chemokine receptor genes. Bar graphs showing relative mRNA expression (arbitrary units, AU) of *CXCR1* (**A**) and *CXCR2* (**B**) in cells isolated from short-term (ST), long-term (LT), and client-owned (CO) dogs at baseline (non-stimulated cells, NS) and after 3 h and 15 h of LPS stimulation. Data are shown as mean results (*n* ≥ 6), and error bars indicate SEM. Statistical analysis was performed by one-way analysis of variance (ANOVA) with Tukey’s multiple comparisons test or the Kruskal–Wallis test followed by Dunn’s multiple comparisons test (* *p* < 0.05, ** *p* < 0.01, *** *p* < 0.001).

Additionally, within-group analyses revealed significant time-dependent changes in *CXCR1* and *CXCR2* expression in short- and long-term shelter dogs, with significantly increased expression at 3 h compared to non-stimulated cells. After 15 h of stimulation, *CXCR1* expression was still significantly elevated in short- and long-term stay dogs (*p* < 0.001, *p* < 0.05, respectively), while *CXCR2* expression remained increased only in short-term stay dogs (*p* < 0.01) (Figure 2A,B).

### 2.5. TFN-α Expression

**Figure 3:**
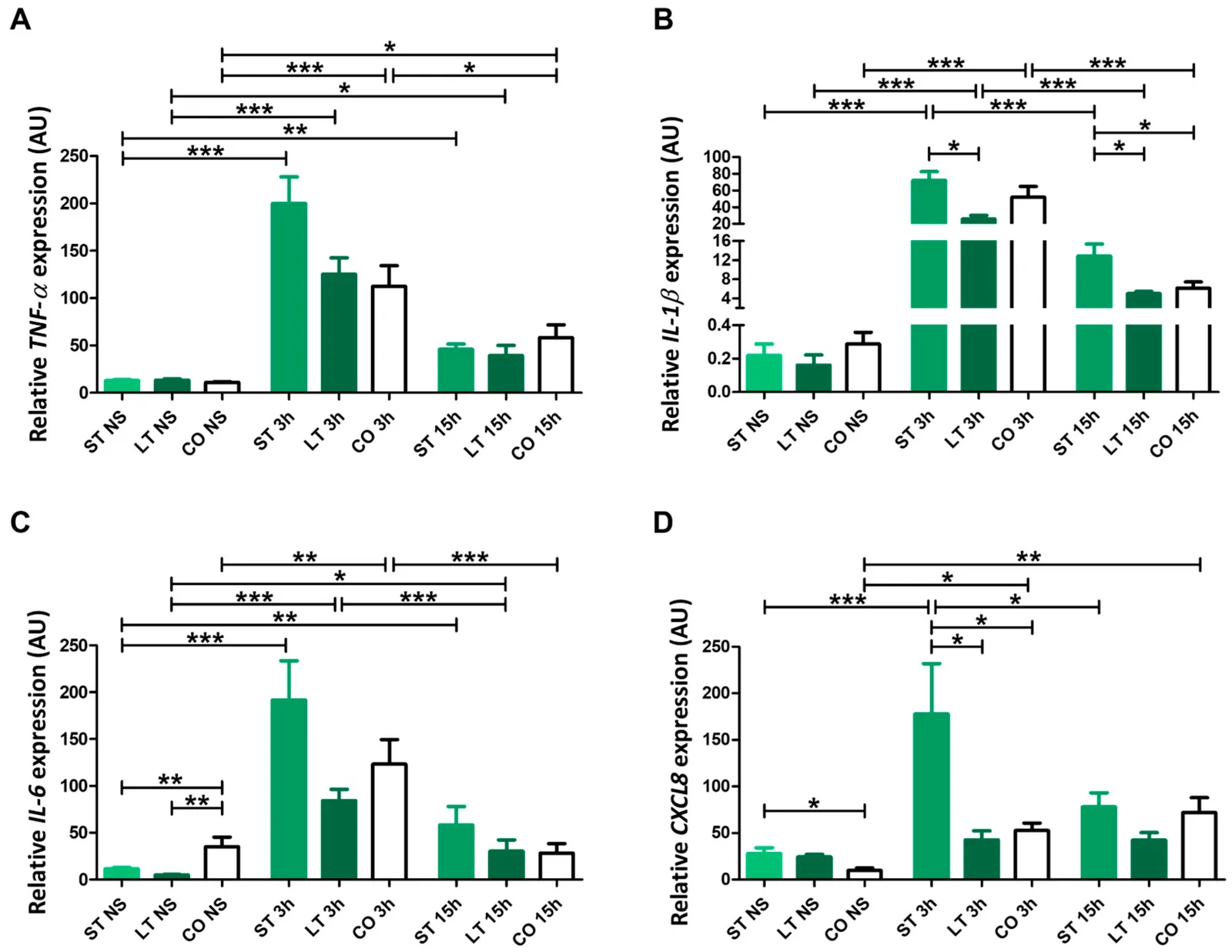
Expression of pro-inflammatory cytokine genes. Bar graphs showing relative mRNA expression (arbitrary units, AU) of *TFN-α* (**A**), *IL-1β* (**B**), *IL-6* (**C**), and *CXCL8* (**D**) in cells isolated from short-term (ST), long-term (LT), and client-owned (CO) dogs at baseline (non-stimulated cells, NS) and after 3 h and 15 h of LPS stimulation. Data are shown as mean results (*n* ≥ 6), and error bars indicate SEM. Statistical analysis was performed by one-way analysis of variance (ANOVA) with Tukey’s multiple comparisons test or the Kruskal–Wallis test followed by Dunn’s multiple comparisons test (* *p* < 0.05, ** *p* < 0.01, *** *p* < 0.001).

*TNF-α* expression did not differ significantly between any of the studied groups at the respective time points. However, significant time-dependent changes were observed within all groups. In short-term stay and long-term stay groups, *TNF-α* expression increased significantly after 3 h of stimulation compared with non-stimulated cells (all *p* < 0.001) and remained elevated after 15 h in comparison to baseline (*p* < 0.01, *p* < 0.05 for short- and long-term, respectively). A similar pattern was observed in client-owned dogs, with a significant increase in *TNF-α* expression after 3 h compared with non-stimulated cells (*p* < 0.001); however, it was followed by a decrease at 15 h (*p* < 0.05). *TNF-α* expression in client-owned dogs remained significantly elevated between non-stimulated cells and the 15 h time point (*p* < 0.05) (Figure 3A).

### 2.6. IL-1β Expression

Baseline *IL-1β* expression did not differ significantly among the studied groups. Following LPS stimulation, significant differences between the groups were observed at both 3 and 15 h. Specifically, *IL-1β* expression was significantly higher in the short-term stay shelter group than in the long-term stay shelter group at both time points (*p* < 0.05). Additionally, after 15 h of stimulation, *IL-1β* expression was significantly higher in the short-term stay shelter group than in the client-owned dog group (*p* < 0.05).

Significant differences were also detected within the groups across the analyzed time points. In all groups, *IL-1β* expression increased significantly at 3 h of LPS stimulation compared with non-stimulated cells and subsequently decreased at 15 h (all *p* < 0.001) (Figure 3B).

### 2.7. IL-6 Expression

*IL-6* expression differed between the studied groups at baseline, with the highest level observed in client-owned dogs compared with both shelter groups (both *p* < 0.01). Following LPS stimulation, no significant differences were observed between the studied groups.

However, significant differences were detected within the groups across the analyzed time points. After 3 h of stimulation, *IL-6* expression increased significantly in the short- and long-term stay groups (both *p* < 0.001) and the client-owned dog group (*p* < 0.01). Subsequently, after 15 h, a significant decrease was observed in long-term and client-owned dogs (both *p* < 0.001). In both shelter groups, however, *IL-6* expression remained significantly higher than in non-stimulated cells (*p* < 0.01, *p* < 0.05 for short- and long-term, respectively). In client-owned dogs, the expression levels returned to values comparable to those observed in non-stimulated cells (Figure 3C).

### 2.8. CXCL8 Expression

Baseline *CXCL8* expression remained significantly higher in the short-term stay dog group compared with the client-owned group (*p* < 0.05). A similar trend was observed after 3 h of stimulation. However, in this case, statistically significant differences were detected between the short-term stay dogs and both the long-term stay and client-owned dogs (both *p* < 0.05).

Differences in *CXCL8* expression depending on stimulation time were also observed within individual groups. In the short-term stay and client-owned dogs, a significant increase in expression was revealed after 3 h of stimulation (*p* < 0.001 and *p* < 0.05, respectively). In the client-owned dogs, *CXCL8* expression remained significantly higher even after 15 h of stimulation compared with non-stimulated cells (*p* < 0.01). In contrast, the opposite effect was observed in the short-term stay group, where expression decreased significantly after 15 h relative to the 3 h time point (*p* < 0.05) (Figure 3D).

### 2.9. TFN-α Secretion

Regarding TNF-α production, no statistically significant differences were detected between the studied groups at baseline. However, after 3 h of LPS stimulation, TNF-α production was significantly higher in the short-term stay shelter dogs compared with both the long-term stay shelter dogs and the client-owned dog groups (both *p* < 0.01). After 15 h of stimulation, this difference remained significant only in comparison with the client-owned dog group (*p* < 0.05).

**Figure 4:**
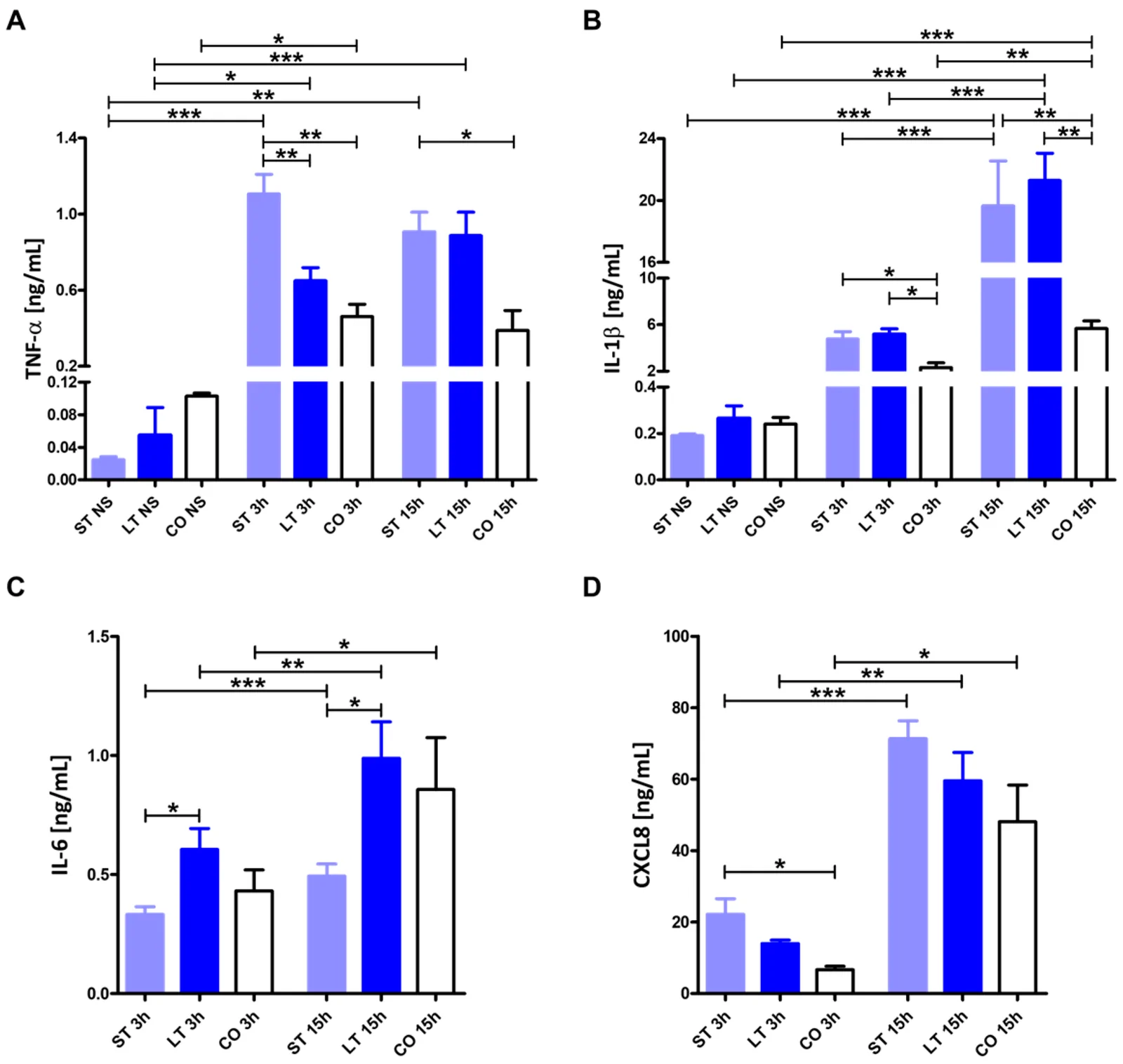
Secretion of pro-inflammatory cytokines. Bar graphs showing cytokine production (ng/mL) of TFN-α (**A**), IL-1β (**B**), IL-6 (**C**), and CXCL8 (**D**) by cells isolated from short-term (ST), long-term (LT), and client-owned (CO) dogs at baseline (non-stimulated cells, NS) and after 3 h and 15 h of LPS stimulation. Data are shown as mean results (*n* ≥ 6), and error bars indicate SEM. Statistical analysis was performed by one-way analysis of variance (ANOVA) with Tukey’s multiple comparisons test and a paired *t*-test (* *p* < 0.05, ** *p* < 0.01, *** *p* < 0.001).

When comparing the different time points within each group, a statistically significant increase in TNF-α production was observed between non-stimulated cells and cells stimulated for 3 and 15 h in both the short-term stay group (*p* < 0.001 and *p* < 0.01, respectively) and the long-term stay group (*p* < 0.05 and *p* < 0.001, respectively). In the client-owned dog group, a statistically significant increase was observed only after 3 h of LPS stimulation (*p* < 0.05) (Figure 4A).

### 2.10. IL-1β Secretion

At baseline, IL-1β secretion did not differ significantly among the studied groups of dogs. Following 3 h of LPS stimulation, IL-1β production was significantly higher in both shelter dog groups compared with the client-owned dogs (both *p* < 0.05). After 15 h of stimulation, this difference persisted and became even more pronounced, reaching a higher level of statistical significance (both *p* < 0.01).

A gradual increase in IL-1β production was observed in all studied groups. This increase was statistically significant between non-stimulated cells and cells stimulated with LPS for 15 h (all *p* < 0.001), as well as between cells stimulated for 3 and 15 h (*p* < 0.001 for both shelter dog groups and *p* < 0.01 for the client-owned dogs) (Figure 4B).

### 2.11. IL-6 Secretion

IL-6 concentrations in serum samples (non-stimulated) were not included in the analysis due to an insufficient amount of biological material. Only a limited number of individual serum samples were analyzed, revealing minimal IL-6 concentrations. Therefore, statistical analyses were performed exclusively on samples collected after 3 and 15 h of LPS stimulation. At both time points, IL-6 levels were highest in the long-term stay group compared with the short-term stay group (*p* < 0.05). Comparisons across the different time points revealed significantly higher IL-6 concentrations at 15 h than at 3 h in all studied groups, with *p* < 0.001 for the short-term stay dogs, *p* < 0.01 for the long-term stay dogs, and *p* < 0.05 for the client-owned dogs (Figure 4C).

### 2.12. CXCL8 Secretion

Serum CXCL8 measurements were not included in the analysis due to insufficient sample volume, and only a limited number of individual serum samples were assessed, yielding minimal detectable concentrations in non-stimulated controls. After 3 h of LPS stimulation, CXCL8 levels were significantly higher in the short-term stay dogs compared with the client-owned dogs (*p* < 0.05), whereas no significant differences between these groups were observed at 15 h. Differences between the analyzed time points followed a pattern consistent across all studied groups, with CXCL8 concentrations differing between 3 and 15 h in the short-term stay dogs (*p* < 0.001), the long-term stay dogs (*p* < 0.01), and the client-owned dogs (*p* < 0.05) (Figure 4D).

## 3. Discussion

The maintenance of immune homeostasis requires leukocytes to rapidly adapt to environmental and microbial stimuli. Neutrophils, which constitute the first line of nonspecific defense against infections, show dynamic changes in the expression of surface receptors, cytokines and chemokine receptors. Such modifications enhance their ability to detect pathogens, migrate to sites of inflammation, and coordinate an effective early immune response [17,18]. Lipopolysaccharide (LPS), a major component of the outer membrane of Gram-negative bacteria, is a potent activator of neutrophils through Toll-like receptor 4 (TLR4) [2]. Previous studies have demonstrated that LPS stimulation may reduce surface TLR4 expression through mechanisms including receptor internalization and endocytosis, thereby modulating cellular responsiveness to prolonged stimulation [19]. Beyond changes in pathogen-recognition receptors, neutrophil activation is accompanied by extensive modulation of surface molecules involved in adhesion, trafficking, and migration [9,13]. Following exposure to inflammatory stimuli such as LPS, neutrophils undergo a coordinated phenotypic shift that facilitates their recruitment from the circulation to sites of infection. This process involves alterations in the expression of adhesion molecules, including the β2 integrin complex CD11b/CD18 (Mac-1) and the selectin CD62L, as well as chemokine receptors such as CXCR1 and CXCR2 [9,10,12]. Together, these molecules coordinate neutrophil adhesion, migration, and inflammatory recruitment. While β2 integrins mediate firm adhesion and transmigration across the endothelium, CXCR1 and CXCR2 regulate chemokine-driven neutrophil chemotaxis toward CXCL8 gradients [10,20]. Both adhesion molecules and chemokine receptors are tightly regulated during inflammatory activation and undergo rapid modulation in response to bacterial products, including LPS [21,22]. Consequently, the expression patterns of these receptors are widely used as indicators of neutrophil activation status and migratory potential during inflammatory responses.

CD11b, a component of the β2 integrin Mac-1 complex, plays a central role in neutrophil adhesion, endothelial attachment, migration, and activation [9,10,23]. Following stimulation, CD11b can be rapidly mobilized from intracellular granules to the cell surface, largely independently of de novo gene transcription [24,25,26]. Therefore, the early functional increase in surface CD11b is primarily driven by granule exocytosis rather than transcriptional upregulation [25]. In the present study, baseline *CD11b* mRNA expression was higher in client-owned dogs compared to shelter dogs, which may reflect differences in integrin mobilization and intracellular storage dynamics under varying environmental conditions. After 3 h of LPS stimulation, short-term stay dogs exhibited a marked increase in *CD11b* expression compared to the other groups, suggesting enhanced early transcriptional responsiveness to antigenic stimulation. Interestingly, after prolonged stimulation (15 h), *CD11b* expression increased significantly within both shelter groups, whereas intergroup differences were no longer evident. This pattern may suggest that although the kinetics of the early response differed between groups, prolonged stimulation ultimately resulted in a comparable transcriptional activation state. This observation is consistent with previous studies indicating that, although early neutrophil responses rely predominantly on rapid mobilization of preformed adhesion molecules, prolonged inflammatory stimulation could additionally induce transcriptional regulation of integrin-associated genes. This interpretation is supported by previous canine studies demonstrating rapid upregulation of CD11b/CD18 on activated neutrophils. Dreyer et al. (1989 and 1995) reported increased surface expression of CD11b/CD18 on canine neutrophils during inflammatory activation, which was accompanied by enhanced endothelial adhesion and migratory capacity [27,28]. Similarly, Nakagaki et al. (2014) demonstrated that stimulation of canine granulocytes induces marked CD11b upregulation, supporting its use as a sensitive marker of granulocyte activation [29]. Together, these findings suggest that the early increase in *CD11b* expression observed in short-term shelter dogs may reflect enhanced responsiveness of adhesion-related pathways. Notably, whereas previous canine studies primarily evaluated surface protein expression, the present study extends these observations by demonstrating time-dependent transcriptional regulation of CD11b following prolonged LPS stimulation.

CD11b and CD18 together form the β2 integrin Mac-1 heterodimer [30]. However, their transcriptional regulation appears to differ substantially. In contrast to *CD11b*, *CD18* expression did not exhibit significant time-dependent changes following stimulation. Instead, a consistent group-dependent pattern was observed across all time points, with client-owned dogs demonstrating significantly lower *CD18* expression compared to both short-term and long-term groups. The persistence of this difference at baseline and after stimulation suggests that *CD18* may undergo minimal transcriptional regulation rather than a dynamic response to in vitro activation. CD18 constitutes the common β-chain shared by multiple β2 integrins and is essential for leukocyte adhesion and transendothelial migration [30]. Its more stable transcriptional profile supports its role as a structurally maintained integrin component. The higher expression observed in dogs from more environmentally exposed groups might indicate an enhanced basal adhesive and migratory potential, possibly reflecting environment-associated immune adaptation. In contrast, the lower *CD18* levels in client-owned dogs could correspond to a comparatively reduced basal activation status under stable environmental conditions. The relatively stable *CD18* transcriptional profile observed in the present study is consistent with previous canine studies indicating that β2-integrin-mediated neutrophil functions are often regulated through changes in integrin availability and surface expression rather than marked alterations in *CD18* expression itself. Studies evaluating CD11b/CD18 expression in canine inflammatory conditions demonstrated that leukocyte activation is associated primarily with the modulation of integrin surface expression and adhesive function, supporting the concept that CD18 serves predominantly as a constitutively maintained structural component of β2 integrins [31,32]. Interestingly, Weiss et al. (2004) reported a decrease in CD18 surface expression on neutrophils from dogs with sepsis and multiple organ dysfunction syndrome (MODS) [33], suggesting post-transcriptional regulation of integrin expression, where changes in receptor abundance at the cell surface may occur independently of transcriptional activity.

In contrast to *CD11b* and *CD18*, *CD62L* expression did not demonstrate statistically significant differences either between groups or across stimulation time points at the mRNA level. Although a non-significant trend toward higher transcript levels in non-stimulated cells was observed in all groups, these changes did not reach statistical significance. CD62L (L-selectin) is primarily regulated at the post-translational level through rapid proteolytic shedding from the cell surface following activation. This process occurs within minutes and can therefore result in substantial changes in surface CD62L expression without requiring changes in *CD62L* transcription [34]. The absence of significant changes in *CD62L* transcript abundance is in agreement with recent findings in canine neutrophils demonstrating that activation-induced regulation of CD62L occurs predominantly through rapid proteolytic shedding rather than transcriptional modulation [35]. Moreover, in the present study, flow cytometric analysis of baseline samples revealed a similar pattern of surface CD62L expression, with short-term shelter dogs exhibiting significantly lower levels than client-owned dogs, despite the absence of significant differences in *CD62L* mRNA expression. This discrepancy between transcript abundance and surface protein expression further supports the involvement of post-transcriptional and post-translational mechanisms in the regulation of CD62L in canine neutrophils. Consequently, substantial alterations in neutrophil adhesive phenotype may occur without detectable changes in *CD62L* mRNA expression, highlighting the limitations of transcript-based assessment for this adhesion molecule.

CXCR1 and CXCR2 are major chemokine receptors involved in neutrophil chemotaxis and migration toward CXCL8 gradients during inflammatory responses. In addition to regulating leukocyte trafficking, these receptors contribute to neutrophil activation and recruitment to sites of infection. Their expression is dynamically modulated during inflammatory stimulation, including in response to bacterial endotoxins [21,36,37]. Previous studies have demonstrated that neutrophil activation may alter CXCR1/CXCR2 expression through mechanisms involving receptor internalization, desensitization, and transcriptional regulation, thereby modulating cellular responsiveness to chemokine signaling [38,39,40]. In the present study, client-owned dogs exhibited significantly higher baseline *CXCR1* and *CXCR2* mRNA expression compared with both shelter groups. These findings may be consistent with differences in the basal transcriptional state of circulating neutrophils, although the contribution of environmental exposure and previous immune stimulation history remains to be determined. Interestingly, after LPS stimulation, significant increases in *CXCR1* and *CXCR2* expression were observed predominantly in shelter dogs, particularly after 3 h of stimulation. These findings may indicate potential differences in the regulation of chemokine receptor-related transcriptional responses among groups following LPS exposure. The observed pattern could also be influenced by differences in receptor regulation kinetics, including the timing of transcriptional activation and subsequent modulation of gene expression. CXCR1 and CXCR2 surface expression in humans may transiently decrease following strong neutrophil activation due to receptor internalization, while later phases of activation may involve compensatory transcriptional responses aimed at restoring receptor availability [40,41,42,43]. Since the present study evaluated mRNA rather than surface protein expression, the transcriptional increases observed after stimulation should not be interpreted directly as increased receptor availability at the cell membrane. Overall, the differential *CXCR1/CXCR2* transcriptional responses observed between shelter and client-owned dogs may indicate group-related differences in neutrophil activation dynamics and chemokine responsiveness following LPS stimulation. However, additional functional studies assessing receptor surface expression and chemotactic activity would be required to determine the biological consequences of these transcriptional alterations, especially since data regarding transcriptional regulation of *CXCR1* and *CXCR2* in isolated canine neutrophils remain limited. Previous canine studies have primarily focused on the biological activity of CXCL8 and its role in neutrophil recruitment rather than on receptor gene expression [44,45]. Therefore, the present findings extend existing knowledge by demonstrating differential transcriptional regulation of *CXCR1* and *CXCR2* in canine neutrophils following LPS stimulation.

CXCL8 is a potent pro-inflammatory chemokine that plays a central role in neutrophil recruitment, activation, and trafficking during acute inflammatory responses. Produced by a variety of immune and non-immune cells, including monocytes, macrophages, endothelial cells, and epithelial cells, CXCL8 promotes directed migration of neutrophils toward sites of infection through interaction with CXCR1 and CXCR2 [46]. In addition to serving as target cells of CXCL8 signaling, canine neutrophils have also been shown to express CXCL8 following inflammatory stimulation, indicating that they may contribute to local amplification of inflammatory responses through chemokine production [47]. In the present study, short-term shelter dogs exhibited significantly higher basal *CXCL8* expression than client-owned dogs. Moreover, a marked increase in *CXCL8* transcription was observed after 3 h of stimulation in both short-term shelter and client-owned dogs, although the response was most pronounced in the short-term group. These findings suggest that neutrophils from recently admitted shelter dogs may exhibit an enhanced early transcriptional response to inflammatory stimulation. The observed *CXCL8* response may be particularly relevant when considered together with the parallel changes in *CXCR1* and *CXCR2* expression. CXCL8 represents the principal chemokine driving neutrophil recruitment through these receptors, and previous canine studies have demonstrated potent chemotactic and activating effects of CXCL8 on canine neutrophils both in vitro and in vivo [44]. Furthermore, CXCL8 has been implicated in neutrophil recruitment during naturally occurring inflammatory diseases in dogs [45]. However, because receptor surface expression and chemotactic activity were not evaluated in the present study, the functional consequences of these transcriptional changes remain to be determined.

TNF-α, IL-6 and IL-1β are among the principal cytokines induced following activation of the TLR4 signaling pathway and play central roles in initiating and amplifying innate immune responses to Gram-negative bacterial components [5]. TNF-α is typically one of the earliest cytokines produced after endotoxin exposure, promoting endothelial activation, leukocyte recruitment, and the production of secondary inflammatory mediators [48]. IL-6 acts downstream of early pro-inflammatory signals and contributes to the development and maintenance of both local and systemic inflammatory responses, whereas IL-1β participates in both the initiation and amplification of inflammatory signaling [49,50,51,52].

In the present study, *TNF-α* expression followed a characteristic pattern in all groups, with a marked increase after LPS stimulation. Notably, no significant differences were observed between the studied groups at any time point. Although *TNF-α* expression decreased between 3 and 15 h in all groups, this decrease was significant only in client-owned dogs, whereas shelter dogs showed no significant change between these time points. This expression profile is consistent with the established role of *TNF-α* as an immediate-early response gene whose transcription is rapidly induced following TLR4 activation and subsequently attenuated through multiple feedback regulatory mechanisms. Similar transient kinetics of *TNF-α* mRNA induction have been reported in canine PBMCs and other mammalian leukocyte populations exposed to endotoxin [53,54]. The similar magnitude and kinetics of *TNF-α* induction observed across groups suggest that the core pro-inflammatory signaling pathways activated by LPS probably remain largely preserved regardless of housing background or duration of shelter stay. A comparable pattern was observed for *IL-6*. Following LPS stimulation, all groups demonstrated a significant increase in *IL-6* expression after 3 h, confirming activation of a robust inflammatory transcriptional response. Subsequently, *IL-6* expression declined after prolonged stimulation, although in shelter dogs, transcript levels remained significantly elevated relative to non-stimulated cells. Previous studies have identified IL-6 as a key downstream mediator of endotoxin-induced inflammation whose expression is regulated by NF-κB and other transcription factors activated following TLR4 engagement [4,53]. The induction observed in the present study therefore corresponds well with the established kinetics of LPS-induced cytokine responses. Notably, the highest expression levels of both *TNF-α* and *IL-6* were observed 3 h after stimulation, supporting their role as early mediators of innate immune activation. Interestingly, client-owned dogs exhibited higher baseline *IL-6* expression than both shelter groups. Although the biological significance of this finding remains unclear, it indicates that differences in the basal transcriptional state of circulating neutrophils may exist between populations despite similar responses to subsequent LPS stimulation. Importantly, these baseline differences disappeared after activation, suggesting that the inducible inflammatory response to endotoxin was comparable and efficient among all groups, despite different housing conditions.

*IL-1β* demonstrated a temporal transcriptional response following LPS stimulation. Although baseline *IL-1β* expression did not differ between groups, significant upregulation was observed in all dogs after 3 h of stimulation, followed by a significant decrease between 3 and 15 h. The rapid transcriptional response of *IL-1β* is consistent with its role as an early mediator of inflammatory responses. Increased *IL-1β* transcription following LPS stimulation has also been reported in activated leukocyte populations and is considered a hallmark of inflammatory activation downstream of TLR signaling [55,56]. Interestingly, the highest *IL-1β* expression was consistently observed in the short-term shelter group at both post-stimulation time points. A similar tendency was apparent for *TNF-α* and *IL-6*, which also reached their highest expression levels 3 h after stimulation in short-term shelter dogs, although these differences did not achieve statistical significance. The overall pattern may reflect differences in the early transcriptional response of neutrophils from recently admitted shelter dogs. However, the biological significance of these observations requires further investigation. This interpretation may be further supported by the concomitant upregulation of *CXCL8*, *CXCR1*, and *CXCR2* observed in the same group during the early phase of stimulation.

While transcriptional profiling provides important insight into cellular activation pathways, cytokine secretion represents the functional outcome of inflammatory stimulation. In the present study, LPS stimulation induced robust production of TNF-α, IL-1β, IL-6, and CXCL8 by canine neutrophils. However, the patterns of cytokine secretion were not always reflected by the corresponding transcriptional responses. Such discrepancies are not unexpected, as cytokine production is influenced by numerous post-transcriptional and post-translational regulatory mechanisms [57]. Hence, transcript abundance alone may not correlate directly with extracellular protein concentrations. These findings highlight that transcriptional activity and cytokine secretion reflect distinct aspects of leukocyte activation. While transcriptional responses characterize the activation state of the cells at the time of sampling, secreted cytokine levels integrate their functional response throughout the stimulation process. Therefore, evaluation of both gene expression and protein production provides a more comprehensive assessment of neutrophil functional responsiveness following inflammatory stimulation.

The increase in TNF-α production observed following LPS stimulation is consistent with previous reports demonstrating that canine neutrophils actively produce TNF-α in response to endotoxin exposure. Li et al. (2013) reported induction of both *TNF-α* gene expression and TNF-α protein secretion in LPS-stimulated canine neutrophils, confirming their active contribution to the early innate inflammatory response [58]. In the present study, *TNF-α* transcript levels did not differ significantly between groups; however, marked differences were observed at the protein level. Following LPS stimulation, neutrophils from short-term shelter dogs produced significantly greater amounts of TNF-α than cells from both long-term shelter and client-owned dogs, particularly during the early phase of stimulation. These findings may reflect additional regulatory mechanisms acting downstream of transcription, as TNF-α production is known to be influenced by post-transcriptional control of mRNA stability and translation, as well as by regulation of cytokine processing and release [59,60]. Additionally, the transient secretion profile observed here, characterized by an early increase following stimulation, is also consistent with the recognized role of TNF-α as an immediate-response cytokine during TLR4-mediated activation [3,58,61].

In contrast to TNF-α, IL-1β secretion increased progressively throughout the stimulation period and remained significantly higher in both shelter groups than in client-owned dogs. The sustained rise in IL-1β production between 3 and 15 h suggests prolonged activation of inflammatory pathways following LPS exposure. To our knowledge, studies specifically evaluating IL-1β secretion by isolated canine neutrophils following prolonged LPS stimulation remain limited. Consequently, direct comparison with previous canine neutrophil studies is difficult. Nevertheless, the present findings indicate that neutrophils derived from shelter dogs produced higher levels of IL-1β following LPS stimulation, thereby expanding the current understanding of functional responses of canine neutrophils.

The results obtained for IL-6 demonstrated only partial agreement between transcriptional and protein-level responses. Although baseline *IL-6* expression was highest in client-owned dogs, the greatest cytokine production following stimulation was observed in long-term shelter dogs. Both shelter dog groups maintained elevated *IL-6* transcript levels after 15 h of stimulation. However, IL-6 expression is also known to be regulated post-transcriptionally through mechanisms affecting mRNA stability and translational efficiency [62,63], indicating that transcript abundance alone may not fully predict cytokine secretion. To date, data regarding IL-6 production have focused on whole blood, peripheral blood mononuclear cells, or in vivo inflammatory models. Studies using canine whole blood and PBMC cultures have consistently demonstrated robust IL-6 induction following LPS stimulation, supporting its role as a key systemic inflammatory mediator in dogs. For example, endotoxin stimulation of canine blood-derived leukocyte populations leads to marked IL-6 production, reflecting activation of inflammatory pathways in vitro [53,64,65,66,67,68]. Therefore, the present study provides additional evidence that canine leukocytes can contribute substantially to IL-6 production during prolonged endotoxin stimulation, extending previous observations.

Among the analyzed cytokines, CXCL8 demonstrated the strongest agreement between transcriptional and protein-level responses. Short-term shelter dogs exhibited both higher *CXCL8* mRNA expression and increased CXCL8 secretion during the early phase of stimulation. This finding is consistent with previous studies demonstrating that activated canine neutrophils are capable of expressing and secreting CXCL8 following inflammatory stimulation. Bazzocchi et al. (2003) reported induction of CXCL8 expression in canine neutrophils following stimulation with Wolbachia surface protein, while Li et al. (2013) demonstrated increased CXCL8 production in LPS-activated canine neutrophils [47,58]. Given the established role of CXCL8 as a potent chemoattractant and activator of canine neutrophils, increased production of this chemokine may contribute to amplification of local inflammatory responses [69,70]. The higher CXCL8 response observed in short-term shelter dogs therefore appears to reflect both enhanced transcriptional activation and increased functional cytokine production.

Taken together, the cytokine secretion data indicate that environmental background may influence not only transcriptional responsiveness but also the functional output of stimulated canine leukocytes. Importantly, the relationship between mRNA expression and cytokine secretion differed among individual cytokines. Whereas CXCL8 showed similarity between transcriptional and protein-level responses, TNF-α and IL-1β demonstrated differences between transcript abundance and cytokine secretion. These findings highlight the complexity of inflammatory regulation in canine neutrophils and emphasize the importance of integrating transcriptional and functional analyses when evaluating innate immune responsiveness. The present study identified differences in the transcriptional and functional responses of canine neutrophils following LPS stimulation. These differences are likely influenced by multiple factors, among which housing background may be one contributing factor. While all groups retained a strong capacity to mount an innate immune response, differences were observed in the kinetics and magnitude of both gene expression and cytokine production, indicating that environmental context may shape neutrophil responsiveness. Distinct patterns were particularly evident in pathways associated with inflammatory activation, leukocyte adhesion, and chemokine signaling. Although the core LPS-induced inflammatory response was preserved across groups, variability in the temporal dynamics of selected mediators suggests differential regulation of early and sustained immune activation. Importantly, transcriptional changes were not always directly reflected at the protein level, highlighting the relevance of post-transcriptional and post-translational regulatory mechanisms in controlling neutrophil function. The duration of housing under different conditions may represent one of the factors contributing to the observed differences in immune responses. Protopopova et al. (2016) suggested that dogs recently admitted to new environments are likely to experience temporary stress and adaptation processes, which may transiently influence leukocyte activation profiles [14]. In contrast, animals maintained under prolonged exposure conditions may undergo adaptation of the immune response. Dogs maintained in stable home environments provide a comparative reference population reflecting baseline immune reactivity under relatively controlled conditions. These differences in environmental exposure history may therefore contribute to the observed heterogeneity in neutrophil responses.

Our study is also associated with some limitations. Although considerable effort was made to ensure the greatest possible homogeneity of the study groups by applying strict inclusion criteria, studies involving companion animals inevitably include a degree of biological variability related to factors such as previous pathogen exposure or genetic background. These variables could not be fully controlled and may have contributed to the observed variability in immune responses. Although the experimental design was focused on neutrophil responses, the isolated cell population consisted of peripheral blood leukocytes. To minimize the potential influence of monocytes, cells were cultured on adherent plates, allowing rapid monocyte adhesion and subsequent collection of the non-adherent fraction for downstream analyses. Moreover, the selected transcriptional markers and functional assays were chosen to predominantly reflect neutrophil activation. Nevertheless, a minor contribution of other leukocyte populations cannot be completely excluded. A more comprehensive characterization of immune status would also benefit from the assessment of peripheral blood leukocyte subsets and a broader panel of inflammatory biomarkers. Finally, the inflammatory response was evaluated exclusively following LPS stimulation, which models bacterial challenge. Future studies incorporating additional stimuli, together with broader immunophenotyping, as well as functional assays such as phagocytosis measurements, would further expand the understanding of canine innate immune responses under different environmental conditions.

Overall, the findings indicate that environmental context may represent one of several factors associated with differences in innate immune responses in dogs. The combined evaluation of transcriptional activity and cytokine secretion offers a more comprehensive understanding of leukocyte biology than either approach alone. This integrative perspective may be useful for future studies aiming to assess immune status and its relationship to environmental stimuli in canine populations. Moreover, the present findings provide a basis for further investigations into canine immune responses, including longitudinal studies and complementary functional approaches aimed at better understanding the dynamics and biological significance of immune adaptation in different environmental contexts.

## 4. Materials and Methods

### 4.1. Animals and Material Collection

The study comprised 36 clinically healthy dogs with an optimal Body Condition Score (BCS) [71,72]. Animals were fed a similar nutritionally balanced diet and had free access to water. All dogs were routinely vaccinated and received antiparasitic prophylaxis, and none had received any medications prior to blood collection. All females were in anestrus with no confirmed pregnancy. Each dog underwent a clinical examination performed by a licensed veterinary surgeon, and blood samples were collected during routine health checkups after obtaining written informed consent from the owner. Blood collection was performed in the morning, within the same time window for all dogs, following a 12 h fasting period. Samples were drawn from the cephalic vein and collected into EDTA-K3 anticoagulant tubes and clotting tubes (both supplied by FL Medical, Torreglia, Italy). All animals underwent basic hematological and biochemical testing, with the parameters within reference values.

The first group comprised 10 client-owned (CO) mixed-breed dogs (4 males, 6 females), aged between 1.5 years and 6 years and 4 months, living in home environments.

The second group included mixed-breed dogs, housed in the same shelter under uniform environmental conditions, which included regular daily walks and human interaction. All dogs from the shelter group came from a single location, allowing for reduced environmental variation. The dogs were between 2 and 6 years old. Shelter dogs were further subdivided into two groups based on the duration of their stay. The short-term group (ST, short-term stay) included 15 dogs (7 males, 8 females) that had resided in the shelter for up to 6 months. The long-term group (LT, long-term stay) comprised 11 dogs (6 males, 5 females) with a length of stay ranging from 6 months to 2 years. This classification approach is similar to the one used by Raudies et al. (2021) [73] and to that of our previous studies [19].

### 4.2. Canine Peripheral Blood Leukocyte Isolation

Whole blood samples were diluted with sterile phosphate-buffered saline (PBS, Sigma-Aldrich, Taufkirchen, Germany) at a 1:2 ratio and carefully layered onto a density gradient medium (Lymphoprep, Stemcell Technologies, Vancouver, BC, Canada). Density gradient centrifugation was performed using SepMate PBMC isolation tubes (Stemcell Technologies, Vancouver, BC, Canada) at 800× *g* for 10 min at room temperature (RT). Next, the isolated cells were washed twice with PBS supplemented with 2 mM EDTA (Sigma-Aldrich, Taufkirchen, Germany) and 2% FBS (Thermo Fisher Scientific, Waltham, MA, USA). Remaining erythrocytes were removed by a 4 min incubation with ACK lysing buffer (Thermo Fisher Scientific, Waltham, MA, USA) at RT. Cells were subsequently washed with sterile PBS and assessed using an automated cell counter (Countess II Automated Cell Counter, Thermo Fisher Scientific, Waltham, MA, USA). Cell viability was evaluated by 4% Trypan blue staining (Thermo Fisher Scientific, Waltham, MA, USA). The isolated cells were resuspended in a culture medium consisting of RPMI-1640 GlutaMAX™ medium supplemented with 10% FBS, 1% sodium pyruvate, 1% nonessential amino acids, 0.1% HEPES, and 1% antibiotics: penicillin and streptomycin (all from Thermo Fisher Scientific, Waltham, MA, USA). Cells were collected at three time points: immediately after isolation (non-stimulated cells, termed NS) and following 3 and 15 h stimulation with lipopolysaccharides (LPS) from *Escherichia coli* O127:B8, suitable for cell culture, and γ-irradiated (Sigma-Aldrich, Taufkirchen, Germany). For stimulation, cells were seeded at a density of 2 × 10^6^ cells/mL in 24-well plates (Corning, New York, NY, USA) in 2 mL of cell medium (described above) and activated with 50 μg/mL of LPS (concentration based on preliminary experiments performed in our laboratory). At each time point, cells were taken and used for real-time PCR analysis, and supernatants (serum for NS, culture medium for 3 and 15 h) were collected for ELISA. All experiments included a minimum of 6 biological replicates per animal group.

### 4.3. RNA Isolation, cDNA Synthesis, and Real-Time PCR Analysis

Cells were washed with sterile PBS and centrifuged at 300× *g* for 8 min. The resulting cell pellets were resuspended in 100 µL of lysis buffer, and total RNA was extracted using the RNAqueous™-Micro Total RNA Isolation Kit (Invitrogen, Thermo Fisher Scientific, Waltham, MA, USA) according to the manufacturer’s instructions. Genomic DNA was removed by DNase treatment, and RNA yield and purity were assessed using a NanoDrop 2000 spectrophotometer (NanoDrop Technologies, Wilmington, DE, USA). Reverse transcription was performed on 1 µg of total RNA using the High-Capacity FG RNA-to-cDNA Kit (Applied Biosystems, Thermo Fisher Scientific, Waltham, MA, USA). Quantitative real-time PCR analysis was carried out with SYBR Green Select Master Mix (Applied Biosystems, Thermo Fisher Scientific, Waltham, MA, USA) following the manufacturer’s protocol on an AriaMx Real-Time PCR System (Agilent Technologies, Santa Clara, CA, USA). All analyses were performed in three technical replicates for each biological sample. The primers used in our study were reported previously: *TNF-α* [74], *IL-6* [53], *IL-1β* [75], *CXCL8*, *CXCR1* [36], *CXCR2* [76], *CD11b* [77], *CD18* [78], and *CD62L* [79]. The *RPS19* gene was used as a non-regulated housekeeping reference for normalization of the target gene expression. The relative mRNA expression was calculated using the comparative Ct method [80] as 2^−ΔCt^ (ΔCt  =  Ct_target_ − Ct_reference_). The sequences of all primers used for qPCR are listed in Table 1.

### 4.4. ELISA

The concentrations of TNF-α, IL-1β, IL-6, and CXCL8 were evaluated in serum and cell medium. All samples were centrifuged (500× *g* for 15 min, 300× *g* for 6 min, respectively), and the resulting supernatant was collected and stored at −20 °C. Measurements were performed using DuoSet ELISA Ancillary Reagent Kit 2 (#DY008B), Canine TNF-alpha DuoSet ELISA (#DY1507), Canine IL-1β DuoSet ELISA (#DY3747), Canine IL-6 DuoSet ELISA (#DY1609), and Canine IL-8/CXCL8 DuoSet ELISA (#DY1608) (all from R&D Systems, Minneapolis, MN, USA) according to the manufacturer’s recommendations. The absorbance was measured with an Epoch Microplate Spectrophotometer (BioTek Instruments, Inc., Winooski, VT, USA) using Gen5 version 1.11.4 software (BioTek Instruments, Inc., Winooski, VT, USA). All analyses were performed in three technical replicates for each biological sample.

### 4.5. Extracellular Staining and Flow Cytometry Analysis

For extracellular staining, isolated PBMCs were washed twice and resuspended in 100 µL of sterile FACS buffer (PBS supplemented with 2% FBS). Fc Receptor Binding Inhibitor (eBioscience, Thermo Fisher Scientific, Waltham, MA, USA) was applied for 20 min to reduce nonspecific binding. To assess surface expression, cells were stained with mouse anti-human CD62L-PE (clone FMC46) antibody (Bio-Rad, Hercules, CA, USA). After incubation, the cells were washed twice with FACS buffer, centrifuged at 300× *g* for 4 min, and resuspended in 200 µL of FACS buffer for analysis. Flow cytometry analyses were conducted using a BD FACS Aria II flow cytometer (Becton Dickinson, Heidelberg, Germany). Each time, a minimum of 50,000 events were recorded. The obtained data were analyzed with FlowJo 7.6.1 software (TreeStar Inc., Ashland, DE, USA). Neutrophils were gated based on their size and granularity using forward-scatter (FSC) and side-scatter (SSC) parameters. Only singlets were included in the analysis.

### 4.6. Statistical Analysis

Statistical analysis was performed using GraphPad PrismTM 5.0 (GraphPad Software Inc., San Diego, CA, USA). Normality was assessed using a Kolmogorov–Smirnov (KS) test. Comparisons between multiple groups were performed using one-way analysis of variance (ANOVA) with Tukey’s multiple comparison test, the Kruskal–Wallis test followed by Dunn’s multiple comparisons test, and a paired *t*-test, as indicated in figure legends. A *p*-value < 0.05 was considered statistically significant. Symbols represent a significant difference between the indicated groups, as follows: * *p* < 0.05; ** *p* < 0.01; *** *p* < 0.001.

## Figures and Tables

**Table 1:** Sequences of primers used in real-time PCR.

Gene	Starter Sequence (F—Forward, R—Reverse)
*TNF-α*	F: 5′-CCAAGTGACAAGCCAGTAGC-3′ R: 5′-TCTTGATGGCAGAGAGTAGG-3′
*IL-6*	F: 5′-CCCACCAGGAACGAAAGAGA-3′ R: 5′-CTTGTGGAGAGGGAGTTCATAGC-3′
*IL-1β*	F: 5′-GAGGTTCCAATGTGAAGTGC-3′ R: 5′-CCTGTAACTTGCAGTCCACC-3′
*CXCL8*	F: 5′-CTTCCAAGCTGGCTGTTGCTC-3′ R: 5′-TGGGCCACTGTCAATCACTCTC-3′
*CXCR1*	F: 5′-GATCGTCTCGCTCCTGAAGG-3′ R: 5′-CAGTGCCTCTTCTGGGTCAG-3′
*CXCR2*	F: 5′-TCATCTTTGCTGTCGTGCTC-3′ R: 5′-TGTGGAAGAAGCCCAGAATC-3′
*CD11b*	F: 5′-GAGTCTGACGATTCCACTAATG-3′ R: 5′-GTTTATGCTGCAGCTGCTA-3′
*CD18*	F: 5′-CGCAGAAAGTGACGCTCTAC-3′ R: 5′-CCGGAAGGTCACATTGAA-3′
*CD62L*	F: 5′-GATTTCAACAGGACCAGCAGCA-3′ R: 5′-CCGCAGGGTCCACAAGTAAGA-3′

## Data Availability

The original contributions presented in this study are included in the article. Further inquiries can be directed to the corresponding author.

## Supplementary Materials

The following supporting information can be downloaded at https://www.mdpi.com/article/10.3390/ijms27188112/s1.
