# Supplementary material for: Functional and Transcriptional Responses of Neutrophils from Dogs Living Under Different Housing Conditions Following In Vitro Lipopolysaccharide Stimulation
Source: Int J Mol Sci. 2026 Sep 11;27(18):8112. doi: 10.3390/ijms27188112 (PMC13607381; doi:10.3390/ijms27188112)
Supplement: Supplementary file 1 [file ijms-27-08112-s001.zip › ijms-4429292-supplementary.pdf]

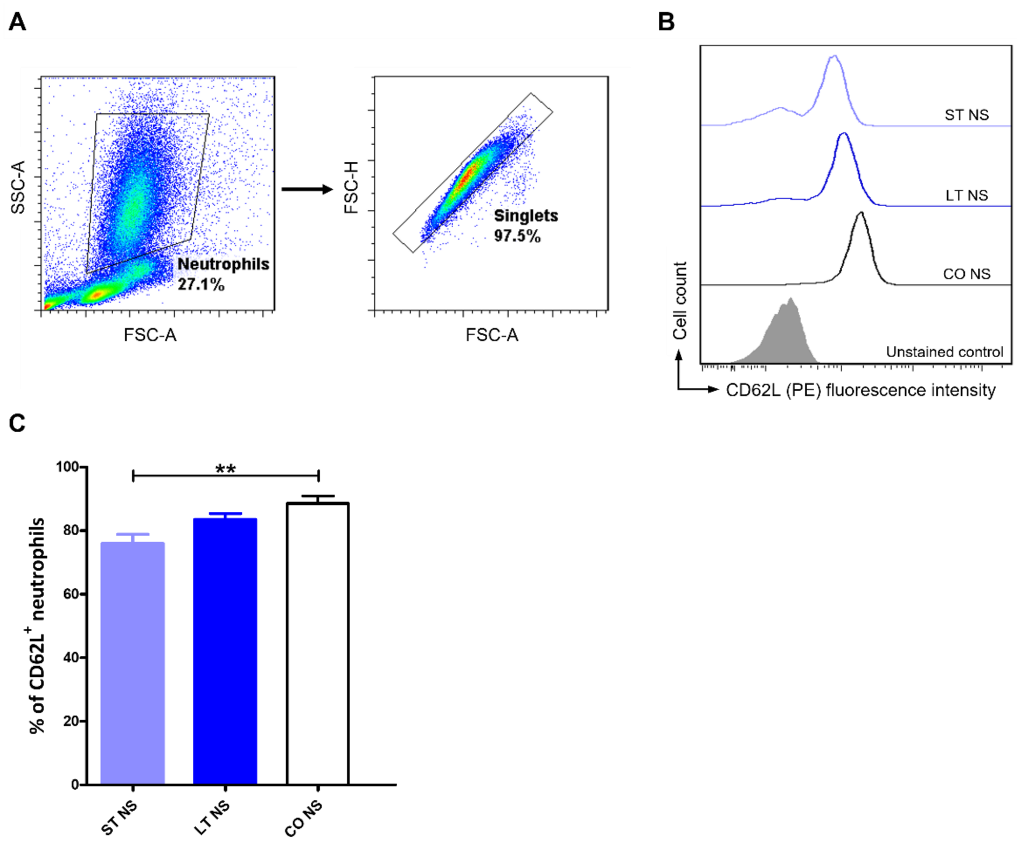

Supplementary Figure S1. Surface expression of CD62L. (**A**) Gating strategy for flow cytometry analysis. Neutrophils were gated based on FSC and SSC. Only singlets were included in further analysis. (**B**) Representative histograms of CD62L (PE) fluorescence intensity on the surface of neutrophils isolated from short-term (ST), long term (LT), and client-owned (CO) dogs at baseline (non-stimulated cells, NS). (**C**) Bar graph showing percentage of CD62L$^+$ neutrophils. Data are shown as mean results ($n \geq 6$), and error bars indicate SEM. Statistical analysis was performed by one-way analysis of variance (ANOVA) with Tukey's multiple comparisons test (**$p < 0.01$).
